# Two-electron spin correlations in precision placed donors in silicon

**DOI:** 10.1038/s41467-018-02982-x

**Published:** 2018-03-07

**Authors:** M. A. Broome, S. K. Gorman, M. G. House, S. J. Hile, J. G. Keizer, D. Keith, C. D. Hill, T. F. Watson, W. J. Baker, L. C. L. Hollenberg, M. Y. Simmons

**Affiliations:** 1 Centre of Excellence for Quantum Computation and Communication Technology, School of Physics, University of New South Wales, Sydney, NSW 2052 Australia; 2 Centre of Excellence for Quantum Computation and Communication Technology, School of Physics, The University of Melbourne, Parkville, VIC 3010 Australia

## Abstract

Substitutional donor atoms in silicon are promising qubits for quantum computation with extremely long relaxation and dephasing times demonstrated. One of the critical challenges of scaling these systems is determining inter-donor distances to achieve controllable wavefunction overlap while at the same time performing high fidelity spin readout on each qubit. Here we achieve such a device by means of scanning tunnelling microscopy lithography. We measure anti-correlated spin states between two donor-based spin qubits in silicon separated by 16 ± 1 nm. By utilising an asymmetric system with two phosphorus donors at one qubit site and one on the other (2P−1P), we demonstrate that the exchange interaction can be turned on and off via electrical control of two in-plane phosphorus doped detuning gates. We determine the tunnel coupling between the 2P−1P system to be 200 MHz and provide a roadmap for the observation of two-electron coherent exchange oscillations.

## Abstract (web summary)

Donor impurities in silicon are promising candidates as qubits but in order to create a large-scale quantum computer inter-qubit coupling must be introduced by precise positioning of the donors. Here the authors demonstrate the fabrication, manipulation and readout of a two qubit phosphorous donor device.

## Introduction

Controlling the interaction strength between two quantum particles lies at the heart of quantum information processing. One must have access to classical control fields that, while tuning the environment of quantum particles, are sufficiently decoupled from them as to not disturb their quantum states^1^. Physical systems ranging from trapped ions^2^, single photons^3^, superconducting circuits^4^ and semiconductor quantum dots^5^ have demonstrated this exquisite level of control. In 1998, Loss and Divincenzo^6^ proposed the use of a controllable exchange interaction in semiconductor quantum dots to perform a two-qubit logic gate. In the same year, Kane^7^ proposed how this could be achieved in donor-based devices. Here, the wavefunction overlap between two electrons on neighbouring donor atoms placed ~20 nm apart is controlled using an exchange gate between them. Harnessing this exchange interaction to perform a universal two-qubit quantum logic gate is the next step for donor-based architectures.

Three approaches exist for donor qubits: a controlled-phase (CZ) gate^8^; the controlled-rotation (C-ROT) gate^9^ and a direct two-electron SWAP operation^10^. While the first two protocols require the use of high frequency microwave fields for electron spin resonance^11^, a direct two-electron SWAP necessitates the ability to turn on and off the exchange interaction between the electrons over orders of magnitude for high fidelity two-qubit operations. Notably, while the extent of a single donor wavefunction is well understood^12–14^, modelling the exchange coupling between two donor electrons is more complex^12–17^ due to multi-valley interference effects^18^. To this end, a critical challenge for donor-based architectures is to know the distance required between the donors in order to turn the exchange interaction on and off with external gates^15,16^.

To date, two main methods for donor placement in silicon exist: ion implantation^19^ and atomic manipulation via scanning-tunnelling-microscopy (STM) hydrogen lithography^20^. Despite much success in accessing randomly placed donor spins, ion implantation has yet to demonstrate donor placement precision below ~6 nm, while STM lithography has demonstrated donor placement down at the atomic scale^21^.

In this paper we use STM lithography that allows both the precision placement of donor atoms for direct and independent spin-measurement of electrons near a readout structure and, most importantly, the control of the exchange interaction between them. We measure the anti-correlated spin states that arise due to the formation of two-electron singlet-triplet states as a function of their wavefunction overlap, which is controlled by in-plane detuning gates. By observing the onset of these anti-correlated spin states as a function of detuning pulse voltage and time, we estimate the magnitude of tunnel coupling between the two donor qubits, and provide a roadmap towards coherent exchange gates for future devices.

## Results

### Independent spin readout of a 2P−1P system

**Fig. 1:**
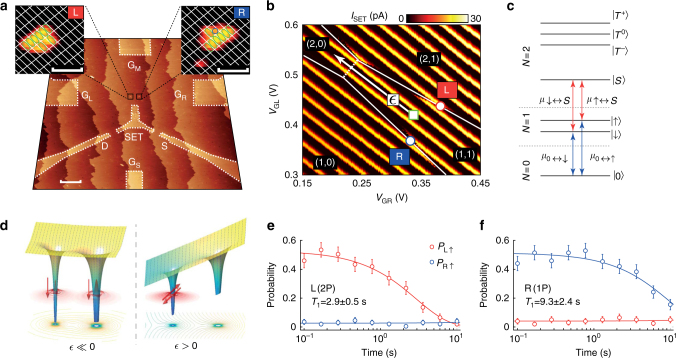
Two qubit 2P−1P device with independent sequential readout. **a** An STM micrograph of a precision placed two spin-qubit donor device showing the lighter coloured lithographic outline where the hydrogen mask has been removed. Two spin-qubits, L and R, are separated by 16 ± 1 nm and sit equidistant at 19 ± 1 nm away from a larger readout structure which serves as both an electron reservoir and single-electron-transistor (SET) charge sensor with source (S) and drain (D) reservoirs and gates {G_L_, G_M_, G_R_, G_S_}; the scale bar is 20 nm. The insets show close-up STM micrographs of L and R where the green (blue) circles show fully (half) desorbed silicon dimers. White lines indicate the silicon dimer rows and the scale bars are 2 nm. **b** Current through the SET charge sensor as a function of *V*_GL_ and *V*_GR_ at the (1,1)–(2,0) charge transition. Electron spin readout is performed at the SET breaks (solid white lines, where tunnelling of qubit electrons to or from SET can occur) at the red and blue circles for L and R respectively. The approximate wait position for spin relaxation measurements is shown by the green square and the detuning axis between (1,1)–(2,0), $\epsilon$, is indicated by the white arrow. The dashed white line indicates where electrons can tunnel between qubit sites, i.e. where $\epsilon$ = 0. **c** The relevant electrochemical potentials in a magnetic field for spin readout of L (red arrows) and R (blue arrows). **d** A schematic representation of the controllable exchange interaction in a 2P−1P donor spin-qubit system. For detuning $\epsilon \ll 0$ the electrons are in the (1,1) charge configuration and the spins are independent. For $\epsilon$ > 0 the ground state (2,0) charge configuration is the two-electron singlet state. **e**, **f** Independent spin readout of L (R) demonstrated by spin relaxation, when the electron on R (L) is deterministically loaded with $\left| \downarrow \right\rangle$. In each case the qubit initially prepared as spin down shows no decay behaviour indicating that the readout is independent at this detuning position, i.e. the exchange is negligible at the readout positions. All measurements were performed with *B*_z_ = 2.5 T

In the original Kane proposal an exchange gate between the donors was suggested to directly tune the exchange coupling between the qubits^7^. Recent tight binding simulations have shown that it is difficult to tune the exchange energy in a 1P−1P donor configuration using such a gate^22^. Instead, it has been proposed that the exchange energy could be tuned over five orders of magnitude^22^ by confining electrons in an asymmetric 2P−1P configuration and by utilising ‘tilt’ control using two opposing detuning gates rather than a central J-gate; see Fig. 1a. Motivated by these predictions with estimates for the required inter-donor separation, in this paper we demonstrate the ability to control exchange coupling in donor-based qubits at the (1,1)–(2,0) charge region using a 2P−1P donor system.

The device, shown in Fig. 1a, was patterned using STM hydrogen lithography. The qubits-L and -R (left and right) are composed of two donors and one donor respectively, determined by examining the size of the lithographic patches (insets in Fig. 1a) as well as their measured charging energies^23,24^ (see Supplementary Figs. 1, 6). Three gates {G_L_, G_M_, G_R_} control the electrostatic environment of the qubits which are tunnel coupled to a larger readout structure made up of approximately 1000 P atoms which serves as a single-electron-transistor (SET) charge sensor. The SET quantum dot is operated with a source-drain bias of 2.5 mV, has a charging energy of ~6 meV and is controlled predominantly via gate G_S_. All data in this paper was taken in a dilution refrigerator with a base temperature of ~100 mK (electron temperature ~200 mK).

Figure 1b shows the charge stability map of the 2P−1P device. Current peaks running diagonally correspond to charge transitions of the SET island. Two sets of breaks in the SET current peaks are observed with different slopes and correspond to electron transitions from either L or R to the SET island. An avoided crossing (triple-point) between these two transitions (dashed white line) indicates the region where electrons can tunnel between L and R, in this case at the (1,1)–(2,0) charge transition. Only one more charge transition corresponding to L is observed at lower gate voltages leading to the assignment of the charge regions.

The direct measurement of anti-correlated electrons hinges upon the ability to independently measure their spin states^25^. To measure the spin of R we employ an energy-selective tunnelling technique^23^ where the electrochemical potential of the single-electron transition from the 1 → 0 charge state is split by the Zeeman energy in a static magnetic field *B*_z_; see blue arrows in Fig. 1c. Whether the electron is able to tunnel to the SET reservoir therefore depends on its spin state, i.e. the readout is a spin-dependent unloading mechanism from the qubit to the SET. This readout technique is employed for the electron at R because the (1,1) region for this qubit borders the 1 → 0 charge states.

For L we use a variant of this method, first reported Watson et al.^26^. The charge transition for this qubit borders the 1 → 2 charge states, but because the chemical potential from the one-electron spin-up and -down states to the two-electron singlet state are also split by the Zeeman energy, a similar readout method is allowed (red arrows in Fig. 1c^25^). In this case, we utilise a spin-dependent loading mechanism from the SET to L. The combination of these two distinct readout techniques avoids the need to pulse over large voltages in order to reach the (1,1)–(2,0) charge transition. Both readout methods are equivalent and give rise to a current ‘blip’ through the SET which is used to discriminate between spin-up and -down electrons. The average readout fidelity of spin-up and -down are estimated to be 96.2 ± 1.1% and 97.6 ± 2.1% for qubit-L and -R respectively (see Supplementary Figs. 2, 3 and Table 1 for full analysis).

Importantly, the readout of each electron must be completely independent of the spin-state of the other. That is to say, the exchange energy at the detuning-position where readout is performed must be vanishingly small, such that no spin flip-flops occur during the readout window. This is demonstrated in Fig. 1e, f. For these measurements we prepare one of two states,

1 $$\rho _{ \downarrow \uparrow } = \frac{{\left| { \downarrow \uparrow } \right\rangle \left\langle { \downarrow \uparrow } \right| + \left| { \downarrow \downarrow } \right\rangle \left\langle { \downarrow \downarrow } \right|}}{2},\rho _{ \uparrow \downarrow } = \frac{{\left| { \uparrow \downarrow } \right\rangle \left\langle { \uparrow \downarrow } \right| + \left| { \downarrow \downarrow } \right\rangle \left\langle { \downarrow \downarrow } \right|}}{2}.$$

where $\left| {i,j} \right\rangle$ indicates the spin state *i* and *j* on qubit-L and -R respectively. Loading spin-down for one qubit is performed deterministically as a result of the spin readout protocol. Spin-up cannot be loaded deterministically; instead a random mixture of spin up and down is loaded by plunging the qubit far below the SET fermi-level. After initialisation we pulse inside the (1,1) charge region midway between the two readout positions (green square in Fig. 1b) and wait for up to 10 s for the randomly loaded electron spin to decay to spin down. Sequential spin-readout of L and then R is performed, in that order, to minimise the effect of the shorter *T*_1_ of qubit-L. The spin-up fractions show relaxation of the qubit initially loaded with random spin, with *T*_1_ times measured to be 2.9 ± 0.5 s and 9.3 ± 2.4 s for electrons on L and R respectively at *B*_z_ = 2.5 T. Importantly, the electron initially loaded as spin-down shows no significant spin-up fraction during this time, demonstrating that at these readout positions there is no significant spin−spin interaction over ~10 s.

### Controllable exchange of precision placed donors

The realisation of a two-qubit logic gate hinges on the ability to controllably turn on and off an interaction between quantum particles. We show this here by pulsing towards the (1,1)–(2,0) charge transition where an exchange interaction between the two electrons arises as a consequence of the Pauli-exclusion principle^27^. The Hamiltonian is given by *H*_ex_ = *J***S**_L_ ⋅ **S**_R_, where **S**_L_ and **S**_R_ are the left and right electron spin vectors and *J* is the strength of the exchange interaction^5^. The magnitude of *J* is given by the energy difference between the symmetric and anti-symmetric two-electron states $\left| {T^0} \right\rangle = \left( {\left| { \uparrow \downarrow } \right\rangle + \left| { \downarrow \uparrow } \right\rangle } \right){\mathrm{/}}\sqrt 2$ and $\left| S \right\rangle = \left( {\left| { \uparrow \downarrow } \right\rangle - \left| { \downarrow \uparrow } \right\rangle } \right){\mathrm{/}}\sqrt 2$ respectively. Similar to gate defined quantum dots, it has been shown that the exchange between donors can also be parameterised in terms of the tunnel coupling and detuning between the (1,1) and (2,0) charge states^28^,

2 $$J(\epsilon ) = \frac{\epsilon }{2} + \sqrt {t_{\mathrm{c}}^2 + \left( {\frac{\epsilon }{2}} \right)^2} ,$$

where $\epsilon$ is the detuning and *t*_c_ is the tunnel coupling (such that *J*(0) = *t*_c_). The detuning axis $\epsilon$ is applied along *V*_GL_ = −0.9 *V*_GR_ (along the SET Coulomb blockade) and is shown by the white arrow in Fig. 1a. It effects a tilting from the (1,1) towards the (2,0) charge state, shown schematically in Fig. 1d. The detuning energy, $\epsilon$, is related to the applied gate voltage *V*_GL_ via the lever arm $\alpha _\epsilon$ = 0.071 eV/V, such that $\epsilon$ = $\alpha _\epsilon$ *V*_GL_.

**Fig. 2:**
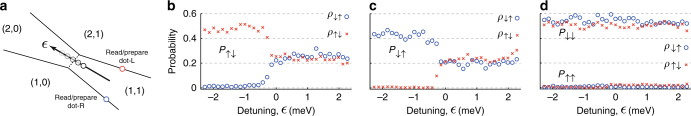
Controllable exchange interaction between precision placed donor-based spin qubits. **a** We prepare a random spin on one qubit and deterministically spin-down on the other near the readout positions in the (1,1) charge region shown by the red and blue circles. After moving into the (1,1) region equidistant between the readout positions for 1 ms (start of arrow), a 50 ms pulse is applied along the detuning axis shown by the black arrow to the positions marked by the black circles. Subsequent pulses are applied to perform spin readout on both qubits. **b**–**d** The probabilities of the joint two-spin outcomes from sequential spin-readout of L and R plotted against the detuning energy, $\epsilon$. For the initially prepared state *ρ*_↓↑_ the blue circles show the outcome of two-electron spin readout which is performed at approximately $\epsilon$ = −7 meV detuning in the (1,1) charge region where exchange is negligible (see Fig. 1). The red crosses show the equivalent data set for an initially prepared state *ρ*_↑↓_

We start by initialising either state from Eq. (1) by loading one qubit randomly and deterministically down on the other, and subsequently apply a 50 ms pulse along the axis $\epsilon$ to control the strength of the exchange interaction^5^, shown by the open black circles in Fig. 2a. This time is long enough to allow for a significant exchange interaction, but much shorter than any electron spin relaxation such that readout is not hindered. Upon pulsing back into the (1,1) charge region we perform independent spin readout of L and then R. In addition to the single spin outcomes for each qubit we also determine the joint probabilities *P*_*ij*_ for *ij* ∈ {↑↑, ↑↓, ↓↑,↑↑}, as shown in Fig. 2b–d.

In the case where *ρ*_↑↓_ is initialised, after pulsing to $\epsilon \ll 0$ we observe *P*_↑↓_ ~ 0.5 and *P*_↓↑_ ~ 0, indicating no spin flip-flops have occurred during the 50 ms pulse duration. However, anti-correlated spins can be seen in Fig. 2b, c as we pulse closer to the (1,1)–(2,0) charge transition, at $\epsilon$ = 0 where both *P*_↑↓_ and *P*_↓↑_ → 0.25. Furthermore, we see that both *P*_↑↑_ and *P*_↓↓_ remain constant at approximately 0 and 0.5 respectively as they represent populations of the triplet states $\left| { \uparrow \uparrow } \right\rangle$ and $\left| { \downarrow \downarrow } \right\rangle$ and are not subject to the exchange interaction. Statistical analysis (see Supplementary Fig. 4) of these results indicates a correlation coefficient of *ϕ* = −0.243 ± 0.028 with a *p*-value $\ll 0.01$ for 0 < $\epsilon$ < 2.4 meV, demonstrating the presence of statistically significant spin anti-correlations in this region.

### Estimate of inter-donor exchange coupling

**Fig. 3:**
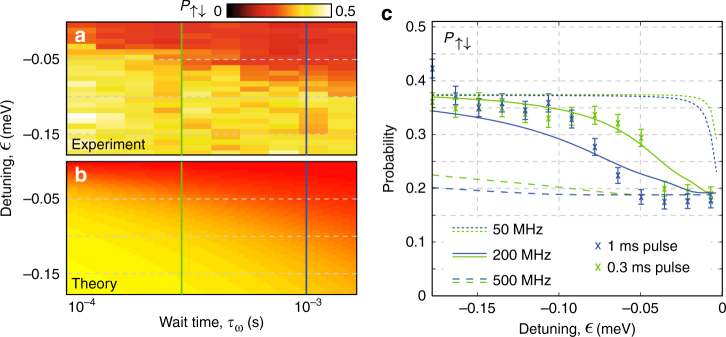
Experimental estimation of tunnel coupling. Starting with *ρ*_↑↓_, the measured probability *P*_↑↓_ (**a**) and the theoretical prediction (**b**) as a function of pulse wait time and detuning position. For the model we have used a value of *t*_c_ = 200 MHz. **c** Solid green and blue curves show theoretical predictions for wait times of 0.3 and 1 ms respectively (corresponding cuts shown in **a**). Blue and green crosses show measurements for these wait times. The dashed and dotted lines show the theoretical predictions for tunnel coupling values of *t*_c_ = 500 and 50 MHz respectively

To ascertain the value of the inter-dot tunnel coupling, *t*_c_, we repeat the same pulsing scheme as above while modifying the detuning pulse duration from 0.1 to 2 ms and compare our results to a spin-level theoretical model; see Fig. 3. The quantum mechanical behaviour of a donor-based two-qubit system is described by the following terms in the Hamiltonian:

3 $$\begin{array}{l}H_{{\mathrm{ze}}} = \gamma _{\mathrm{e}}{\bf{B}} \cdot \left( {{\bf{S}}_{{{\rm L}}} + {\bf{S}}_{{{\rm R}}}} \right),\\ H_{{\mathrm{zn}}} = \gamma _{\mathrm{n}}{\bf{B}} \cdot \left( {{\bf{I}}_{{\rm{n}}_{{\rm{L1}}}} + {\bf{I}}_{{\rm {n}}_{{\rm{L2}}}} + {\bf{I}}_{{\rm{n}}_{\rm{R}}}} \right),\\ H_{{\mathrm{hf}}} = A_{\mathrm{L}}{\bf{S}}_{\rm{L}} \cdot \left( {{\bf{I}}_{{\rm{n}}_{{\rm{L1}}}} + {\bf{I}}_{{\rm{n}}_{{\rm{L2}}}}} \right) + A_{\mathrm{R}}{\bf{S}}_{\rm{R}} \cdot {\bf{I}}_{{\rm{n}}_{\rm{R}}},\\ H_{{\mathrm{ex}}} = J{\bf{S}}_{\rm{L}} \cdot {\bf{S}}_{\rm{R}},\end{array}$$

where *H*_ze_ and *H*_zn_ are the electron and nuclear Zeeman energies, with *γ*_e_ ≈ 28.024 GHz/T and *γ*_n_ ≈ 17.235 MHz/T gyromagnetic ratios respectively^29^. The hyperfine term, *H*_hf_ is separated into two components as it has been predicted that the hyperfine constants will be different for varying donor cluster configurations^23,30^. Here for simplicity we assume the bulk-like value of *A*_L_ = *A*_R_ = *A* = 117.53 MHz^29^ and define the static field to be ${\bf{B}} = \left( {0,0,\left| {B_{\rm z}} \right|} \right)$. We numerically calculate the time evolution of the density matrix via a fourth-order Runge−Kutta method with the inclusion of relevant decoherence channels (see Supplementary Fig. 5).

**Fig. 4:**
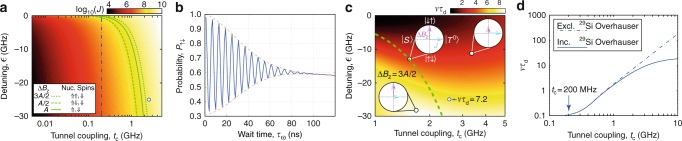
Theoretical predictions for the observation of coherent exchange oscillations. **a** The value of exchange energy, *J*, as a function of tunnel coupling, *t*_c_ and detuning, $\epsilon$. The boundary separating the two-electron product states with singlet-triplet states occurs where the difference in magnetic field between the two qubits, Δ*B*_z_ is equal to the exchange energy. For donor-based systems Δ*B*_z_ is dominated by the donor hyperfine strength, and is equal to *A* for a 1P−1P system (solid green line), and can take the two values *A*/2 or 3*A*/2 for a 2P−1P system (dashed and dotted green lines respectively) dependent on the nuclear spin orientation (examples shown in inset). We assume the bulk 1P value for the hyperfine, *A* = 117.53 MHz. The dashed blue line indicates the values of *J* accessible for the current device with *t*_c_ ~ 200 MHz. **b** Theoretical prediction of coherent exchange oscillations for a 2P−1P device in natural silicon with tunnel coupling *t*_c_ = 2.5 GHz. The two-electron state is initialised as $\left| { \uparrow \downarrow } \right\rangle _{}^{}$ at a point where the exchange energy is negligible, and subsequently a non-adiabatic detuning pulse is applied to $\epsilon$ = −25 GHz (circle marker in **a**). We have assumed voltage noise equivalent to 850 MHz along the detuning axis, $\epsilon$ (obtained from measurements) as well as a single electron $T_2^ \ast = {55\,{\mathrm {ns}}}$ measured in previous works^32^. From this result an oscillation frequency *ν* and dephasing time *τ*_d_ are extracted. **c** The product of oscillation frequency, *ν* and dephasing time, *τ*_d_ as a function of tunnel coupling and detuning. The green dashed line represents the boundary between product and singlet-triplet eigenstates of the two-electron system. The Bloch sphere cross sections indicate the relative magnitudes of Δ*B*_z_ (purple) and *J* (blue) in different regions. **d** Theoretical prediction of *ντ*_d_ along the line Δ*B*_z_ = *J* as a function of tunnel coupling for a 2P−1P double quantum dot. Solid (dashed) line shows results including (excluding) the ^29^Si Overhauser field

For the theoretical data shown in Fig. 3b we prepare the initial state *ρ*_↑↓_ from Eq. (1) and simulate non-adiabatic pulses to detuning positions for varying pulse durations, *τ*_w_. For this simulation we use a tunnel coupling, *t*_c_ = 200 MHz, assume dynamic P nuclear spins as well as a single-spin dephasing time of $T_2^ \ast = {55\,\mathrm {ns}}$ due to the constantly fluctuating Overhauser field of the ^29^Si nuclear spins. The equivalent measured data set is shown in Fig. 3a with cuts at 0.3 and 1 ms shown in Fig. 3c and compared with the theoretical predictions for *t*_c_ = 50, 200 and 500 MHz. From these results we can estimate the tunnel coupling within an order of magnitude accuracy to be *t*_c_ ~ 200 MHz for this device. Following Eq. (2), this result places an equivalent bound on the achievable exchange energy *J* < 200 MHz inside the (1,1) charge region (see Fig. 4a).

In ref. ^22^ the authors investigated multiple different 2P intradot configurations, and found that disorder at the lattice-site level had little effect on the final exchange energy. They showed that the exchange energy for a 2P−1P system with a 15 nm separation was tunable over five orders of magnitude for electric field strengths $- 2 < \left| {\bf{E}} \right| < 2\,{\mathrm {MV/m}}$ at the donor sites. The voltage applied to the in-plane gates in our device amount to a potential difference of ~ 100 mV between *V*_GL_ and *V*_GR_ at the (1,1)–(2,0) inter-dot transition. From an electrostatic model of our device we estimate $\left| {\bf{E}} \right| = 0.49 \pm 0.10\, {\mathrm {MV/m}}$ at the donor sites. Our estimate of *t*_c_ = 0.2 GHz (which is equal to *J* at $\epsilon$ = 0) is within an order of magnitude of the theoretical prediction for *J* in a 2P−1P system given this $\left| {\bf{E}} \right|$^22^.

### Requirements for coherent control of exchange

In this final section we investigate the potential for achieving coherent exchange between two electrons confined to donors in natural silicon. Based on Eq. (2) the plot in Fig. 4a shows the obtainable exchange energies, *J*, as a function of detuning and tunnel coupling, where the vertical blue dashed line shows *t*_c_ = 0.2 GHz for our device. Importantly, the boundary where the difference in magnetic field at the two qubit sites Δ*B*_z_ is equal to the exchange energy *J*, separates the two-electron product eigenstates and singlet-triplet eigenstates. For donor qubits Δ*B*_z_ is dominated by the phosphorus nuclear-spin hyperfine, *A*. The exact value of Δ*B*_z_ varies depending on the number of donors at each qubit site and their nuclear spin orientations: For a 2P−1P device with random nuclear spin configurations Δ*B*_z_ fluctuates between 3*A*/2 or *A*/2 with a 1:3 ratio.

It can be seen from Fig. 4a that for the device studied here there exists only a small range in detuning (approximately 10 μV in gate voltage) over which one could implement coherent exchange oscillations inside the (1,1) charge region (negative $\epsilon$). When one takes into account any voltage noise on gates (which influences $\epsilon$ and ultimately *J*) this makes the operation of coherent oscillations challenging^31^. Indeed, in this particular device we measured gate RMS voltage noise of 50 μV from shot to shot, equivalent to detuning noise of *δ*$\epsilon$ = 850 MHz, indicating that pulsing repeatedly to the same exchange energy would not be possible for $\epsilon$ < 0. For the same reasons, charge noise also destroys coherence when adopting the approach to pulse $\epsilon$ > 0. We carried out experiments with pulses down to 10 ns for both $\epsilon$ < 0 and $\epsilon$ > 0 but were unable to observe a coherent exchange phenomenon.

Figure 4b shows the predicted number of exchange oscillations (~15) that would be observed in a device with *t*_c_ = 2.5 GHz after pulsing to a detuning $\epsilon$ = −25 GHz (circle marker in Fig. 4a). Conversely, using the same model we estimate that a noise floor of <6 μV (~100 MHz in detuning or ~50 mK, much lower than the electron temperature) would be required to observe the signature of coherent oscillations in the present device. Note that in addition to the realistic detuning noise we have also included the effect of a constantly fluctuating ^29^Si Overhauser field expected in natural silicon^32^ as well as randomised P donor nuclear spins of the donor atoms themselves.

From these simulations we can extract the frequency of oscillations, *ν* as well as the dephasing time *τ*_d_, allowing us to determine the figure of merit *ντ*_d_ as a function of tunnel coupling and detuning pulse position; see Fig. 4c. Interestingly, the product *ντ*_d_ only becomes significant beyond the boundary Δ*B*_z_ = 3*A*/2 for values of *t*_c_ > 2 GHz, providing a lower bound on the required tunnel coupling for coherent control. Figure 4d gives *ντ*_d_ as a function of tunnel coupling for a detuning pulse to the boundary Δ*B*_*z*_ = 3*A*/2. These results indicate that at high tunnel coupling, the observation of exchange oscillations will ultimately be hindered by the presence of the fluctuating ^29^Si Overhauser field. In the case where qubits exist in a spin vacuum, as in ^28^Si, only charge noise is relevant and *ντ*_d_ can be seen to increase monotonically as a function of tunnel coupling (dashed line in Fig. 4d).

## Discussion

In summary, we have demonstrated a controllable exchange interaction resulting in two-electron spin anti-correlations on precision placed 2P−1P donors qubits in Si using in-plane ‘detuning’ gates. The results are consistent with the exchange interaction behaviour expected at the (1,1)–(2,0) charge transition and represent the first direct measurement of correlated electron spins in donor-based devices. While the small tunnel coupling (0.2 GHz) in the present device prohibited measurement of coherent oscillations, we show our results agree with recent studies^15^ in which much smaller distances than previously predicted are required to achieve a sufficiently large exchange coupling for coherent control. Furthermore, while detuning noise presents a problem for devices with a small tunnel coupling, we theoretically predict that for larger tunnel couplings of *t*_c_ > 2 GHz it can be overcome. Theoretical work on coupled donor systems suggest a separation of 13–14 nm between a 1P−2P system will be required to achieve this magnitude of exchange coupling^22^. Importantly, there is no reason to believe that this small change in donor site separation will lead to a significant reduction in electrical controllability based on previous experimental works^24,25,28,33^. This benchmark for a larger interaction strength between neighbouring donor-based qubits provides the focus for future experiments.

With the atomic precision placement of donors using STM lithography it will be possible to further optimise the inter-donor distance to control the coherent coupling between two donor qubits with order-of-magnitude accuracy^34^. While extensive studies have been conducted for deterministic single P donor incorporation^35^, similar studies will need to be developed to determine the optimal lithographic patch for deterministic 2P incorporation. Crucially, recent theory predicts that the 2P−1P configuration we present in this paper both increases the tunability of the tunnel coupling and at the same time suppresses the ‘exchange fluctuations’ known for two single donors, and may therefore be less sensitive to the exact atomistic donor positions than two coupled single donors^22^. Furthermore, our ability to directly place the donor with <1 nm accuracy along with the reproducible demonstration of high fidelity single-shot spin-readout in multiple devices^25^, bode well for the future scalability of donor qubit quantum computers.

## Methods

### Device fabrication

The device, shown in Fig. 1, was fabricated using scanning-tunnelling-microscopy hydrogen lithography to selectively remove hydrogen from a passivated Si(100) 2 × 1 reconstructed surface. The lithographic mask is subsequently dosed with PH_3_ and annealed (320 °C) to incorporate P atoms into the silicon substrate^36^ with ~1/4 monolayer density (2 × 10^14^/cm^2^) allowing for quasi-metallic conduction in all electrodes^37^.

### Measurement setup

For all electrical measurements, the device was mounted on a high-frequency printed circuit board within a copper enclosure, thermally anchored to the cold finger of a dilution refrigerator with a base temperature of 50 mK. Voltage pulses were applied to gates G_L_ and G_R_ by an arbitrary waveform generator (Agilent 81180A), connected via a bias tee to the gate along with a constant-voltage source. The SET current, *I*_SET_, was amplified and converted into a voltage signal at room temperature, low-pass filtered to 1 kHz bandwidth, and acquired with a fast digitising oscilloscope.

### Data availability

The data that support the findings of this study are available from the corresponding author upon reasonable request.

## Electronic supplementary material

Supplementary Information
