# Supplementary material for: Two-electron spin correlations in precision placed donors in silicon
Source: Nat Commun. 2018 Mar 7;9:980. doi: 10.1038/s41467-018-02982-x (PMC5841377; doi:10.1038/s41467-018-02982-x)
Supplement: Supplementary file 1 — Supplementary Information [file 41467_2018_2982_MOESM1_ESM.pdf]

Supplementary Methods

Charging energy calculation

The charging energy, E_c^a of a quantum dot (QD) a can be calculated by knowing the charging energy of another QD, b which is capacitively coupled to a [1]. They are related through their mutual charging energy, E_m, which is given by,

$$E_\mathrm{m} = \alpha_g^i \delta V_g^i, \tag{1}$$

where α_g^i is the lever arm from gate g to QD i and δV_g^i is the voltage shift of the potential of QD i due to QD j. This value must be the same for both QDs a and b. Therefore, we can write,

$$\alpha_g^a \delta V_g^a = \alpha_g^b \delta V_g^b. \tag{2}$$

In the same manner, E_c^i is given by,

$$E_\mathrm{c}^i = \alpha_g^i \Delta V_g^i, \tag{3}$$

where now, ΔV_g^i is the voltage difference between two charge transitions in the space of gate g. We can eliminate the (usually) unknown lever arms by combing equations Eq.(2) and Eq.(3),

$$\frac{E_\mathrm{c}^a}{\Delta V_g^a} \delta V_g^a = \frac{E_\mathrm{c}^b}{\Delta V_g^b} \delta V_g^b, \tag{4}$$

We now consider the possibility that $E_\mathrm{c}^a \neq E_\mathrm{c}^b$ such that multiple charging events can occur in the voltage range ΔV_g^i. In this case, the measured $\Delta V_g^i$is actually the sum of the true ΔV_g^i, which we denote $\Delta \hat{V}_g^i$ and the number of charging events, n^j of the other QD,

$$\Delta V_g^i = \Delta \hat{V}_g^i + n^j \delta V_g^j. \tag{5}$$

Since we require $\Delta \hat{V}_g^i$ in Eq.(4), we must substitute Eq.(5) in such that it now reads,

$$\frac{E_\mathrm{c}^a}{E_\mathrm{c}^b} = \frac{\delta V_g^b (\Delta V_g^a - n^b \delta V_g^a)}{\delta V_g^a (\Delta V_g^b - n^a \delta V_g^b)}. \tag{6}$$

This is the general form of the equation which relates the two charging energies between QDs a and b. We now look at the specific case where $\Delta V_g^a \gg \Delta V_g^b$. This is the case most commonly seen when we have a large QD used as a charge sensor to measure smaller QDs or single donors (which will necessarily have larger charging energies than the charge sensor QD). For clarity, we switch to the S/D (SET/donor) terminology in place of $a = D$ and $b = S$. If the condition $\Delta V_g^D \gg \Delta V_g^S$ holds then there will be multiple charging events of the SET ($n^S \neq 0$) and exactly zero for the donor ($n^D = 0$) within the voltage ranges ΔV_g^D and ΔV_g^S, respectively. As a result, we can simplify Eq.(6) to,

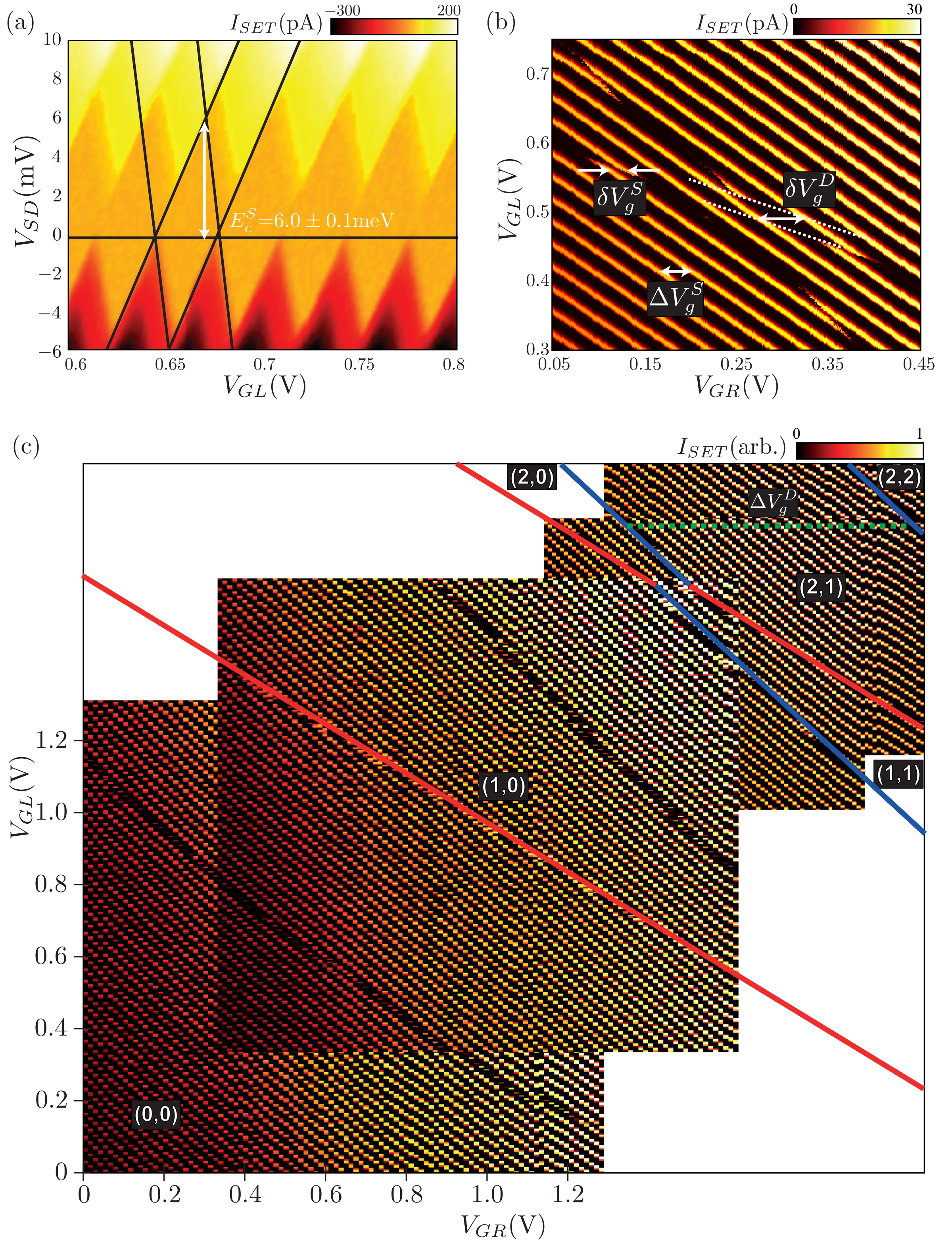

Supplementary Figure 1: **Definition of the parameters used in calculating the charging energy for the two qubits**. **a**, The Coulomb diamonds used to extract the charging energy of the SET, E_c^S. **b,** A charge stability map (V_{GL} vs. V_{GR}) showing the definition of the voltage parameters, δV_g^S, δV_g^D, and ΔV_g^S. **c**, A composite charge stability map showing all the observed charge transitions in our device as a function of V_{GL} and V_{GR}. The four separate maps vary in the middle gate voltage, V_{GM} from $0.1 - 0.7$ V. Red and blue lines show charge transitions of qubits L and R respectively. Other observed transitions are attributed to charge traps in the vicinity of the SET and are not relevant to the experiment. The definition of ΔV_g^D is shown by the green dashed line. The number of SET charge transitions, n^S are counted along this line.

$$E_{\mathrm{c}}^{D} = \frac{E_{\mathrm{c}}^{S}\delta V_g^{S}}{\Delta V_g^{S}}\left(\frac{\Delta V_g^{D}}{\delta V_g^{D}} - n^{S}\right). \quad (7)$$

Supplementary Figure 1 shows the measurement of all required parameters $\{E_{\mathrm{c}}^{S},\ \delta V_g^{S},\ \delta V_g^{D},\ \Delta V_g^{S},\ \Delta V_g^{D}\}$, where we have chosen to measure along the right gate, G_{R}, i.e. $g = \mathrm{R}$. Using Eq.(7) for the $1 \rightarrow 2$ electron transitions for both qubits, we find charging energies of 65 ± 8 and 43 ± 5 meV and for L (2P) and R (1P), respectively. These values are consistent with theoretical [2,3] and previously measured [3,4] charging energies for 2P and 1P donor qubits respectively.

Sequential spin readout of two donor qubits

The top panels of Supp. Fig. 2a and b show close-ups of the current through the SET charge sensor in the region where spin readout is performed on R and L, respectively. The position of the three readout phases, load (L), read (R) and empty (E) are shown by the white circles in these diagrams. As discussed in the main text both readout techniques rely on a spin-dependent tunnelling process [5], this can be seen in the data presented in the bottom panels of Supp. Fig. 2a and b. Here, the average SET current in time is shown as the read voltage is stepped along the axis shown by the white arrow in the upper panels (equivalent to the detuning axis ε in the main text). At certain read voltages a short current blip can be seen to occur at the start of the read phase indicating the spin-up of the electron qubit.

Sequential spin readout of electrons on L and R is carried out using the sequence of pulses shown in Supp. Fig. 2c. The first phase of this sequence is the read phase of qubit-L, after which a pulse is applied to the read out position of qubit-R (positions 1 and 2). Since the readout is independent at these detuning positions i.e. exchange is negligible (as shown in Fig. 1 of the main text) the electron remaining on qubit-L during the read phase of qubit-R has no effect on spin readout fidelity. The following four pulses from 3-6 serve to empty and reload electrons from and to the two qubit sites, these may or may not occur depending on the exact experimental protocol, i.e. whether or not a qubit is being prepared with random spins or deterministically with spin-down. Finally, we pulse to position 7 from which we carry out the exchange pulse as described in Fig. 2a of the main text.

As described in the main text, the mechanisms used for readout at qubit-L and -R differ. For qubit-R, at the (1,0)-(1,1) charge transition, we perform a spin-dependent unloading mechanism and in the case of qubit-L, at (1,1)-(2,1), a spin-dependent loading mechanism is used to discriminate between spin-up and -down states. In a similar vein the loading of random spins is different for each qubit. For qubit-R we plunge below the Fermi level of the SET by approximately 0.35meV (5mV in detuning along the axis ε) loading from (1,0)→(1,1), whereas for qubit-L we unload from the singlet state, (2,1) →(1,1), by plunging *above* the Fermi level by the same detuning.

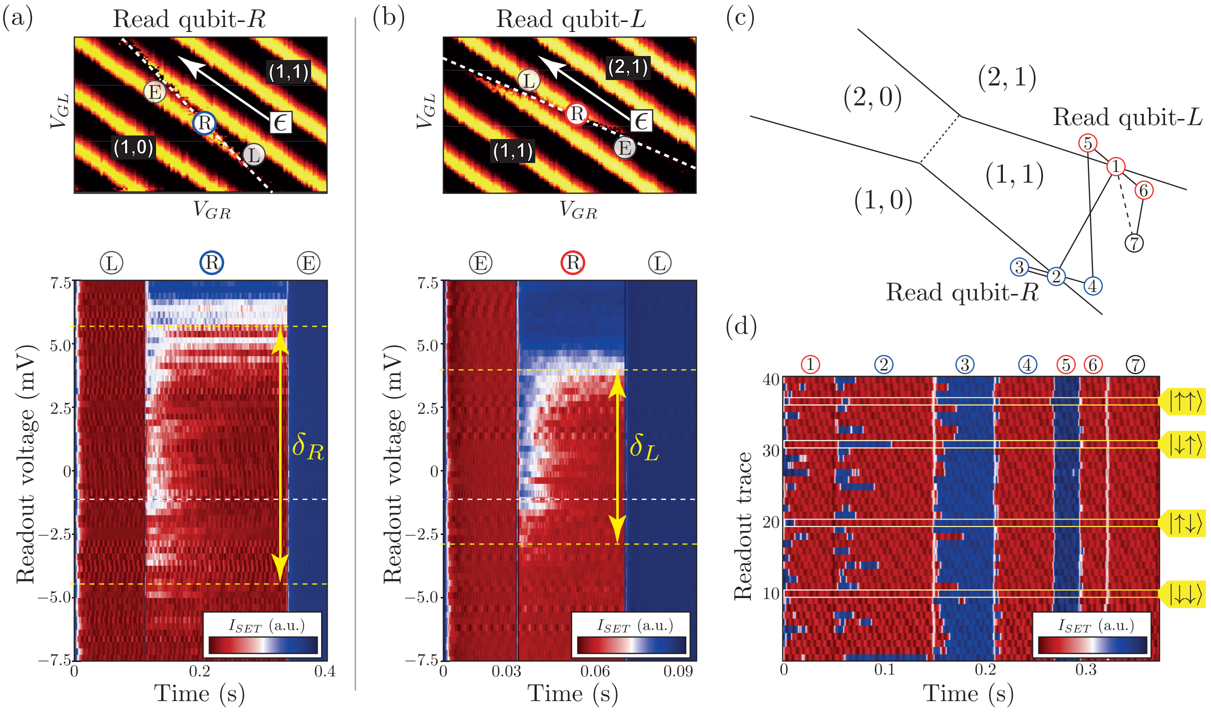

Supplementary Figure 2: **Sequential spin readout of two donors**. **a**, (upper) The three level pulse scheme for electron spin readout of qubit R depicted on the charge stability map at the $1 \rightarrow 0$ charge transition (white dashed line). The approximate positions of the load, read and empty phases (R, L, E) of the three level pulse are shown by the circle markers. Spin readout of the electron at this donor relies on a spin-dependent *unloading* mechanism from the qubit to the SET reservoir at the position marked `R' in the diagram [5]. The read voltage is stepped along the SET Coulomb blockade peak, shown by the white arrow, and is equivalent to ε axis described in the main text. (lower) The average of 200 single-shot SET current traces, I_{SET}, as a function of the read voltage along ε. The voltage at which spin readout is performed during the experiments is shown by the white dashed line. The range over which spin-up electrons can selectively tunnel off of the dot, δ_{R}, is shown by the yellow arrow. All measurements were performed at $B_z = 2.5$ T. **b**, A similar readout method is employed for qubit L. Here, a spin-dependent *loading* mechanism from the $1 \rightarrow 2$ charge state is utilised. **c**, Schematic of the pulsing sequence used to sequentially readout L and R in that order, as well as initialise both qubits with random spin states. The order of spin readout is chosen to minimise the effects of spin relaxation since L has the shorter T_1 time. To initialise spin-down deterministically on either qubit, we skip phases 3,4 for R and/or 5,6 for L. **d**, An example of 40 single shot traces for the sequential spin readout and initialisation of random spins on L and R for the sequence shown in **c**. The two read phases occur at the beginning of each trace. A short `blip' in the SET current indicates the tunnelling of an electron during the read phases (1 and 2) in approximately 50% of the traces. This occurs due to the presence of a spin-up electron on the dot. Example traces for the outcomes {↑↑, ↑↓ , ↓↑, ↓↓} are shown.

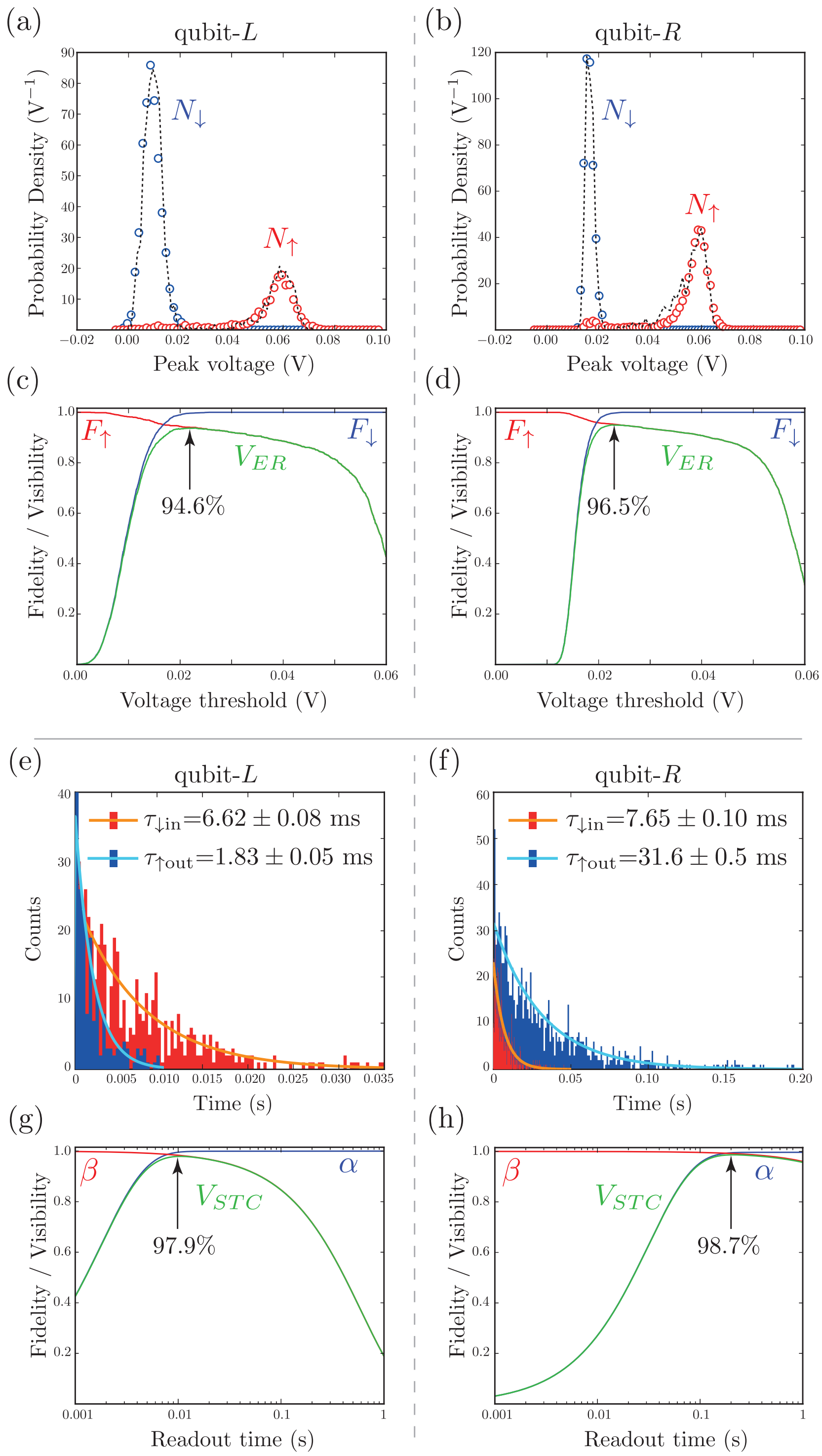

Supplementary Figure 3: **Readout fidelity analysis**. **a-d**, Calculation of electrical readout fidelity, V_{ER}. A simulation of 10,000 SET traces during readout with 50% containing `blips' and with the same signal-to-noise ratio as measured in the experiment, allows for an optimisation of the readout voltage threshold, V_t being where V_{ER} is maximised. Blue and red circles in **a,b** show the simulated spin-up and -down traces respectively, whereas the dashed black line shows the experimental distribution of maximum voltage during the readout phases. The fidelities $F_\uparrow$ and $F_\downarrow$ in **c,d** are calculated using Eq.(8,9) as a function of the readout threshold V_t. **e-h**, Calculation of the spin-to-charge conversion fidelity, V_{STC}. The relevant tunnel times from L and R are measured experimentally from 2000 SET readout traces shown in **e,f**. A rate equation model developed in Refs [6,7] is used to estimate the optimum readout time, Δt, which optimises the successful assignment of a spin-up or -down, α and β respectively. From these individual fidelities the maximum spin-to-charge conversion fidelity, V_{STC} can be estimated for both qubits, shown in **g,h**.

Electrical readout fidelity

The assignment of a spin-up or -down electron from each SET current trace comprises of two separate parts, (i) electrical readout and (ii) spin-to-charge conversion.

The electrical readout involves determining whether a given SET current trace can be assigned as having a `blip' during the read phase i.e. whether during this time the current surpasses a threshold value $I_t$ (see Supp. Fig. 2). From a simulation of 10,000 SET traces 50% of which contain a `blip', and with added white Gaussian noise equivalent the signal-to-noise ratio observed in the experiment (average of $SNR = 17$ dB for readout of both qubits), histograms of peak voltages V_p are generated and shown in Supp. Fig. 3a and b for L and R respectively. Note the use of peak voltage not current due to the use of a current amplifier on the drain of the SET charge sensor.

From these histograms the fidelity of assigning either spin-up or -down ($F_\uparrow$ or $F_\downarrow$) to each current trace is calculated using the following set of equations,

$$F_\uparrow = 1 - \int_{-\infty}^{V_t} N_\uparrow(V_p)dV_p \quad (8)$$

$$F_\downarrow = 1 - \int_{V_t}^{\infty} N_\downarrow(V_p)dV_p, \quad (9)$$

where V_t is the equivalent voltage threshold for I_t after the current amplifier and N_i is the fraction of spin state i. The results are shown in Supp. Fig. 3c and d with the addition of the calculated electrical readout visibility $V_{ER} = F_\uparrow + F_\downarrow - 1$. From this we can determine the optimum voltage threshold, V_t, where V_{ER} is maximised.

Parameter	Qubit-L	Qubit-R
V_t (V)	0.022 ± 0.001	0.016 ± 0.002
Δt (ms)	10.5 ± 0.1	209.0 ± 30.0
$F_\uparrow$ (%)	94.9 ± 0.8	96.8 ± 1.6
$F_\downarrow$ (%)	99.7 ± 0.2	99.8 ± 0.2
α (%)	99.6 ± 0.1	99.5 ± 0.1
β (%)	98.2 ± 0.1	99.1 ± 0.1
V_{ER} (%)	94.6 ± 1.0	96.5 ± 2.0
V_{STC} (%)	97.9 ± 0.1	98.7 ± 0.2
F_M (%)	96.2 ± 1.1	97.6 ± 2.1

Supplementary Table 1: **Experimental parameters for spin readout of qubits L and R.**

Spin-to-charge conversion

Next we determine the optimum length of time for the read phase of the three level readout sequence. During spin-to-charge conversion errors are introduced from three main sources; T_1 relaxation of spin-up electrons; spin-up electrons failing to tunnel to the SET during the designated read time; and spin-down electrons tunnelling to the SET due to thermal excitation. Note that the second and third points apply specifically to the spin dependent unloading mechanism used at R. However, equivalent arguments apply for L in the case of the spin dependent loading mechanism.

Following from the work of Buch [6] and Watson [7] we use a rate equation model to determine the optimum readout time, Δt, based on the probability of a successful assignment of spin-up or -down, α and β respectively. As an input to the model the tunnelling times of spin-up out of the qubit site to the SET and spin-down into the qubit site from the SET, $\tau_{\uparrow out}$ and $\tau_{\downarrow in}$ are shown in Supp. Fig. 3e and f. In addition, the spin-down tunnelling time from the qubit site to the SET, $\tau_{\downarrow out}$ was also measured experimentally to be and 0.61 ± 0.06 s and 25 ± 5 s for qubit-L and -R respectively. We refer the reader to Ref. [6] for further details on this model. The readout time, Δt vs the fidelities α and β are shown in Supp. Fig. 3e-h. Similarly for the electrical readout, the visibility of spin-to-charge conversion is calculated as $V_{STC} = \alpha + \beta - 1$. The optimum readout time is chosen where V_{STC} is maximised. Supplementary Table 1 gives a summary of the fidelity calculations for both qubits, where the final measurement fidelity is given by, $F_M = (\alpha F_\downarrow + \beta F_\uparrow)/2$.

Calculation of lever arms along the detuning axis ε

The lever arm of the gates along the detuning axis ε can be ascertained from the range in read voltage over which tunnelling due to spin-up electrons on the qubits is observed (the so called *spintail*). This range, labelled δ_i in the lower panels of Supp. Fig. 2a and b, is proportional to the Zeeman splitting via the lever arms, α_ε^L and α_ε^R for qubit-L and -R respectively, by

$$\alpha_\varepsilon^i \delta_i = \gamma_e B_z, \tag{10}$$

where $\gamma_e = 28.024$ GHz/T and B_z is the magnetic field. From Supp. Fig. 2 the lever arms were calculated to be $\alpha_\varepsilon^L = 0.041 \pm 0.004$ and $\alpha_\varepsilon^L = 0.030 \pm 0.003$. The sum of these two lever arms represents scaling of the detuning, ε *between* the qubit-L and -R to the gate voltage V_{GL} along this axis.

Statistical analysis of detuning dependent spin correlations

The statistical significance of the anti-correlated spin measurements presented in Fig. 2 of the main text can be ascertained using the ϕ-correlation coefficient (the Pearson correlation coefficient for two binary variables), which is given by,

$$\phi_m = \frac{P_{\uparrow\uparrow}P_{\downarrow\downarrow} - P_{\downarrow\uparrow}P_{\uparrow\downarrow}}{\sqrt{P_{L\uparrow}P_{L\downarrow}P_{R\uparrow}P_{R\downarrow}}}. \tag{11}$$

For perfectly anti-correlated spins $\phi = -1$, however, given our choice of initial states from Eq. 1 in the main text, where one spin is randomly loaded up or down and the other is deterministically loaded with spin-down, the maximum expected value is $\phi = -0.25$. We measure an average of $\phi_m = -0.243 \pm 0.028$ in the detuning range $0 < \varepsilon < 2.4$ meV for both $\rho_{\downarrow\uparrow}$ and $\rho_{\uparrow\downarrow}$, see Supp. Fig. 4a. The statistical significance of these anti-correlations was deduced from the p-value of $\chi^2 = \phi^2/n$, where $n = 1000$ is the number of measurement repetitions. A p-value $\ll 0.01$ is shown in Supp. Fig. 4b over the same range of ε, demonstrating statistically significant two-electron anti-correlated spins.

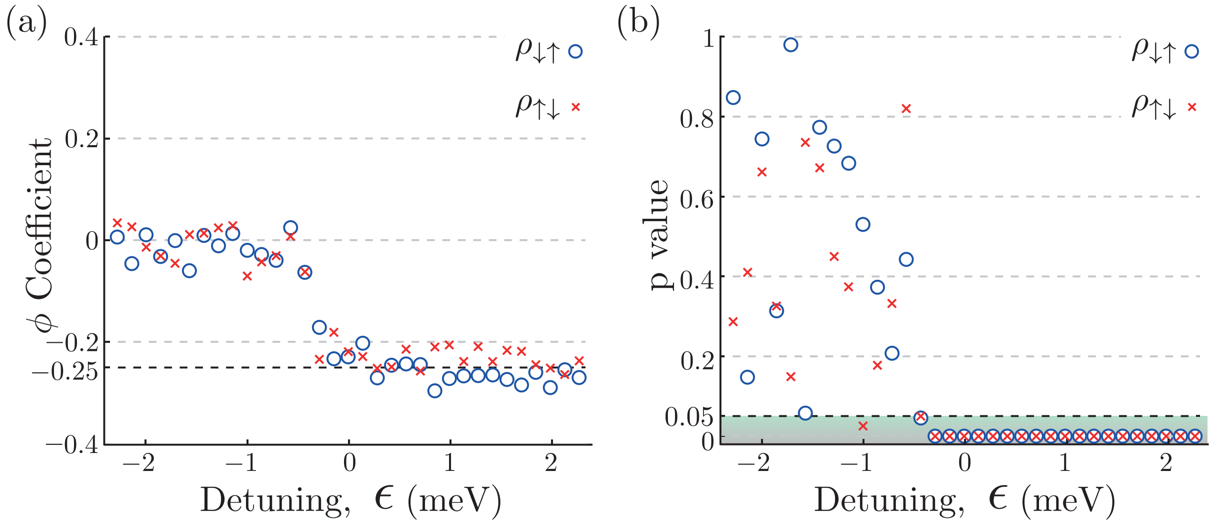

Supplementary Figure 4: **Statistical analysis of detuning dependent spin correlations**. **a**, The ϕ-correlation coefficient defined in Eq.(11) showing the onset of anti-correlated spins as a function of detuning for the initially prepared states $\rho_{\downarrow\uparrow}$ and $\rho_{\uparrow\downarrow}$ given in Eq. 1 of the main text. The expected theoretical value for anti-correlated electron spins is -0.25 (black dashed line). **b**, The p-value of the χ^2 test for this dataset. Data points below a threshold of 0.05 are considered statistically significant.

Modelling the coherent exchange oscillations

Using the same Hamiltonian given in Eq. 3 of the main text we model coherent exchange oscillations as described in Fig. 3 and 4 of the main text. For this proposed experiment, both electron spins are initialised at a large negative detuning position where the exchange is negligible and subsequently pulsed non-adiabatically into a region where the exchange dominates over hyperfine, that is where $J > \Delta B_z$. The spins are allowed to evolve for some time τ_w before being pulsed back to the initial preparation positions, where spin readout can be carried out. In our simulation we replicate this pulse sequence with the inclusion of detuning noise with a Gaussian distribution [8] defined by a standard deviation of 850MHz in detuning energy, based on gate noise measurements of approximately $\delta\varepsilon = 50\mu$V measured in our device. We average over 100 repetitions of the simulation to obtain the final density matrix. In addition to detuning noise, we include a constantly fluctuating Overhauser field which equates to a single electron $T_2^* = 55$ ns measured in previous electron spin resonance experiments in natural silicon [9]. Finally, we average over all eight possible donor nuclear spin configurations for the 2P-1P system, giving us an average representation of the nuclear hyperfine interaction. The detuning pulse sequence is simulated for varying τ_w times, giving rise to the coherent oscillations that can be seen by the green markers in Fig. 3b of the main text.

The form of coherent exchange oscillations, as shown in Fig. 4b of the main text, can also be approximated analytically in the following way. Firstly, the oscillation frequency resulting from the exchange interaction, ν, depends on the relative magnitude of the exchange energy J and the difference in magnetic field between the two qubits ΔB_z. For donor systems, ΔB_z is dominated by the donor nuclear spin orientation resulting in a difference in hyperfine strength between the two qubits, δA. For a particular nuclear spin configuration the frequency ν is given by,

$$\nu = \sqrt{J^2 + \delta A^2}, \tag{12}$$

similarly, the undamped amplitude of these oscillations Λ is given by,

$$\Lambda = \frac{J^2}{\sqrt{J^2+\delta A^2}}. \tag{13}$$

The amplitude is averaged over all nuclear spin configurations but it is assumed that the frequency is dominated by the most common configuration, which in the 2P-1P case is $\delta A = A/2$.

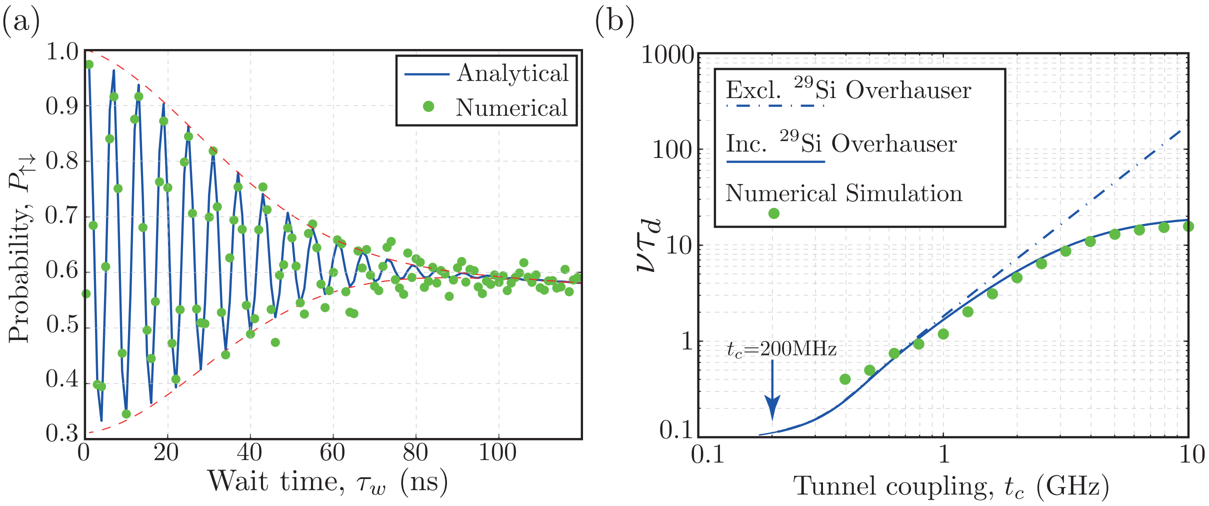

Supplementary Figure 5: **Comparison of analytical and numerical models for coherent exchange oscillations**. **a**, Theoretical prediction of coherent exchange oscillations for a 2P-1P device in natural silicon with tunnel coupling $t_c = 2.5$ GHz and an applied pulse to $\varepsilon = -25$ GHz (circle marker in Fig.4a of main text) for a time τ_w. The two electron state is initialised as $|\uparrow\downarrow\rangle$ at a point where the exchange energy is negligible, and subsequently a non-adiabatic detuning pulse is applied to $\varepsilon = -25$ GHz. We have assumed voltage noise equivalent to 850~MHz along the detuning axis, ε (obtained from measurements) as well as a single electron dephasing time of $T_2^* = 55$ ns measured in previous works [9]. The results for a numerically simulated full quantum model are shown by the green markers, while the blue line gives the predicted curve based on an analytical expression in Eq.(17). **b**, Theoretical prediction of $\tau_d\nu$ along the line $\Delta B_z = J$ as a function of tunnel coupling for a 2P-1P donor qubit system. Solid (dashed) lines show analytical results including (excluding) the ^{29}Si Overhauser field, whilst the green markers are results from a numerical simulation.

As in the numerical case, the dephasing is a combination of detuning noise and an Overhauser field. The standard deviation of the detuning noise $\delta\varepsilon = 50\mu$V can be transformed into an exchange frequency noise $\delta\nu$ by considering the minimum and maximum oscillation frequencies given the $\delta\varepsilon$,

$$\delta\nu = |\nu(\varepsilon + \delta\varepsilon, \delta A) - \nu(\varepsilon - \delta\varepsilon, \delta A)|. \tag{14}$$

Note here we have also averaged ν over all possible nuclear spin orientations which give rise to different values of δA. For a 2P-1P donor dot system, $\delta A = 3A/2$ or $A/2$ with a 1 to 3 ratio. Based on the width, $\delta\nu$, in the frequency domain the resulting dephasing time τ_J is calculated by converting to the time domain and is given by,

$$\tau_J = \frac{1}{\pi \delta \nu}. \quad (15)$$

Note that the decay induced by τ_J is expected to be Gaussian based on the nature of the noise [9]. The total dephasing time τ_d which includes dephasing from the constantly fluctuating Overhauser field is approximated using the formula,

$$\frac{1}{\tau_d} = \frac{1}{\tau_J} + \frac{1}{T_2^*}. \quad (16)$$

Finally, the form of the analytical coherent exchange oscillations is given by,

$$\frac{1}{2} + \frac{[\Lambda e^{-\left(\frac{t}{\tau_J}\right)^2} \cos(2\pi\nu t) + (1-\Lambda)] e^{-\frac{t}{T_2^*}}}{2}. \quad (17)$$

Both the characteristic decay time τ_d and oscillation frequency ν change as a function of the pulse detuning position ε and tunnel coupling t_c, and their product $\tau_d \nu$ gives an indication of the number of observable oscillations. This is plotted in Fig. 3c of the main text as a function of the tunnel coupling and pulse detuning position. In Supp. Fig. 5 we shown a comparison of the analytical expressions derived above against a numerical simulation with equivalent parameters as described in previous section.

Supplementary Discussion

B-field dependence of single-electron T_1 relaxation

Supplementary Figure 6a and b shows the dependence of spin-up fraction of each qubit as a function of wait time for different magnetic field strengths. Both qubits show a B_z^5dependence of the $1/T_1$ relaxation rate as shown in Supp. Fig. 6c, indicating that the relaxation processes are driven by phonon coupling to the electron spins as previously observed [6,9,10]. However, one thing we do have to be careful of is the magnetic field orientation during the measurement. Note that the results for independent readout presented in the main text are shown by square markers in Supp. Fig. 6c at $B_z = 2.5$ T and were obtained with a field orientation of $B_z^{(1)}||[1\bar{1}0]$. The magnetic field dependence of T_1 are shown by the red and blue circular markers and were taken during a different cool down with a field orientation $B_z^{(2)}||[100]$. Since T_1 relaxation times are known to be highly sensitive to the magnetic field orientation [11--13], the difference in the observed T_1 values between the two cool downs can be explained by this.

There are two interesting aspects of the results presented in Supp. Fig. 6c. Firstly, we measure the spin relaxation rate of a 2P donor qubit in this device to be greater than that for the 1P donor case. This is in contrast to recent experimental [14] and theoretical results [15], which show a slower relaxation rate for multi-donor qubits in the one-electron case due to its tighter confining potential [15]. At this time, we do not have a theoretical model to describe this discrepancy, but speculate that it could be due to the specific spatial configuration of the donors inside the 2P cluster. Furthermore, we note that an unexpected anisotropy in T_1 in a magnetic field has recently been observed in donor based systems by the present group and further work is needed to determine whether this can explain the effect observed herein.

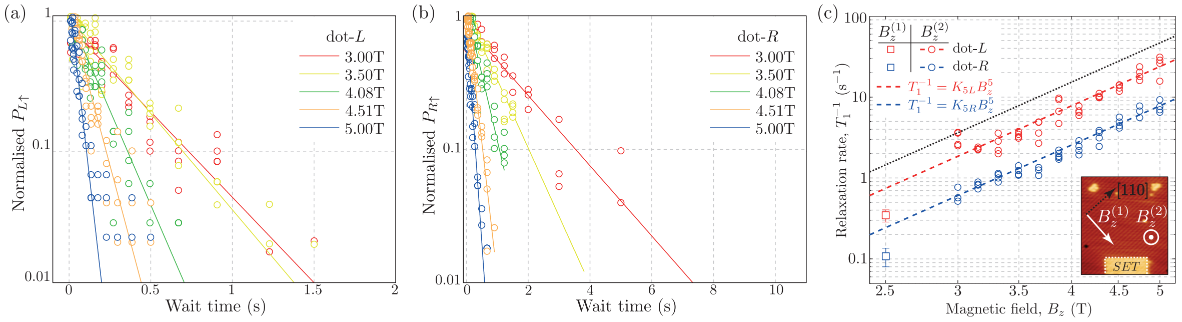

Supplementary Figure 6: **Dependence of donor T_1 relaxation times on magnetic field**. **a**, The normalised probability of measuring $|\uparrow\rangle$ on L as a function of wait time for different values of magnetic field, B_z. Data is plotted with circle markers and fits to exponential decays are given by the solid lines. **b**, An equivalent set of T_1 vs. B_z data for R. **c**, The T_1 relaxation of qubits L and R given in Fig. 1(e and f) of the main text (red and blue squares respectively) was measured at $B_z = 2.5$ T with the orientation $B_z^{(1)}||[1\bar{1}0]$ as shown in the inset. The magnetic field dependence of T_1 relaxation was measured for the field orientation $B_z^{(2)}||[100]$ (separate cool down), and shown by the red and blue circle markers for qubits L and R respectively. Fits to $T_1^{-1} = K_{5i}B_z^{\ 5}$ are shown by the dashed lines, with the prefactor $K_{5L} = 0.0060 \pm 0.0010$ s^{-1}T^{-5} and $K_{5R} = 0.0025 \pm 0.0001$ s^{-1}T^{-5}. Multiple measurements at the same field indicate the spread in T_1 value. For comparison the dotted black line shows the result for a single P donor T_1 relaxation ($K_5 = 0.015$ s^{-1}T^{-5}) measured originally by Morello [9] and confirmed later by Watson [16] for a field orientation $B_z||[110]$.

Secondly, we see that for the two field orientations $B_z^{(1)}$ and $B_z^{(2)}$ (see inset of Supp. Fig. 6c) the 1P relaxation rates are significantly slower than those measured previously [9,16], giving a value of ~22s and ~52s for qubits L and R respectively at B=1.5T. Further work is underway to examine the interplay of the magnetic field and electric field orientation in these Coulomb confined devices.

Supplementary References

[1] S. J. Hile, M. G. House, E. Peretz, J. Verduijn, D. Widmann, T. Kobayashi, S. Rogge, and M. Y. Simmons. Radio frequency reectometry and charge sensing of a precision placed donor in silicon. Appl. Phys. Lett., 107:093504, 2015.

[2] A. L. Saraiva, A. Barna, M. J. Calderon, and B. Koiller. Theory of one and two donors in silicon. J. Phys.: Condens. Matter, 27:154208, 2015.

[3] B. Weber, Y. H. Matthias Tan, S. Mahapatra, T. F. Watson, H. Ryu, R. Rahman, L. C. L. Hollenberg, G. Kilmeck, and M. Y. Simmons. Spin blockade and exchange in Coulomb-con_ned silicon double quantum dots. Nature Nanotech., 9:430--435, 2014.

[4] M. Fuechsle, J. A. Miwa, S. Mahapatra, H. Ryu, S. Lee, O. Warschkow, L. C. L. Hollenberg, G. Klimeck, and M. Y. Simmons. A single-atom transistor. Nature Nanotech., 7:242--246, 2012.

[5] J. M. Elzerman, R. Hanson, L. H. Willems van Beveren, B. Witkamp, L. M. K. Vandersypen, and L. P. Kouwenhoven. Single-shot read-out of an individual electron spin in a quantum dot. Nature, 430:431--435, 2004.

[6] H. Buch, S. Mahapatra, R. Rahman, A. Morello, and M. Y. Simmons. Spin readout and addressability of phosphorous donor clusters in silicon. Nature Commun., 4:2017, 2013.

[7] T. F. Watson. PhD thesis, School of Physics, University of New South Wales, Sydney, Australia, 2015.

[8] L. T. Hall, J. H. Cole, C. D. Hill, and L. C. L. Hollenberg. Sensing of uctuating nanoscale magnetic fields using nitrogen vacancy centers in diamond. Phys. Rev. Lett., 103:220802, Nov 2009.

[9] A. Morello, J. J. Pla, F. A. Zwanenburg, K. W. Chan, K. Y Tan, H. Huebl, M. Mottonen, C. D. Nugroho, C. Yang, J. A. van Donkelaar, A. D. C. Alves, D. N. Jamieson, C. C. Escott, L. C. L. Hollenberg, R. G. Clark, and A. S. Dzurak. Single-shot readout of an electron spin in silicon. Nature, 467:687{691, 2010.

[10] M. Xiao, M. G. House, and H. W. Jiang. Measurement of the spin relaxation time of single electrons in a silicon metal oxide-semiconductor-based quantum dot. Phys. Rev. Lett., 104:096801, Mar 2010.

[11] Hiroshi Hasegawa. Spin-lattice relaxation of shallow donor states in ge and si through a direct phonon process. Phys. Rev.,118:1523--1534, Jun 1960.

[12] Laura M. Roth. g factor and donor spin-lattice relaxation for electrons in germanium and silicon. Phys. Rev., 118:1534--1540, Jun 1960.

[13] Pengke Li, Jing Li, Lan Qing, Hanan Dery, and Ian Appelbaum. Anisotropy-driven spin relaxation in germanium. Phys. Rev. Lett., 111:257204, Dec 2013.

[14] Thomas F. Watson, Bent Weber, Yu-Ling Hsueh, Lloyd C. L. Hollenberg, Rajib Rahman, and Michelle Y. Simmons. Atomically engineered electron spin lifetimes of 30 s in silicon. Science Advances, 3(3), 2017.

[15] Yu-Ling Hsueh, Holger Bu□ch, Yaohua Tan, Yu Wang, Lloyd C. L. Hollenberg, Gerhard Klimeck, Michelle Y. Simmons, and Rajib Rahman. Spin-lattice relaxation times of single donors and donor clusters in silicon. Phys. Rev. Lett., 113:246406, Dec 2014.

[16] T. F. Watson, B. Weber, M. G. House, H. Buch, and M. Y. Simmons. High-fidelity rapid initialization and read-out of an electron spin via the single donor D- charge state. Phys. Rev. Lett., 115:166806, Oct 2015.
